# Influences of obese (*ob/ob*) and diabetes (*db*/*db*) genotype mutations on lumber vertebral radiological and morphometric indices: Skeletal deformation associated with dysregulated systemic glucometabolism

**DOI:** 10.1186/1471-2474-7-10

**Published:** 2006-02-01

**Authors:** Katherine M Burkemper, David R Garris

**Affiliations:** 1 Division of Cell Biology and Biophysics, School of Biological Sciences, University of Missouri-Kansas City, Kansas City, Missouri 64110 USA

## Abstract

**Background:**

Both diabetes and obesity syndromes are recognized to promote lumbar vertebral instability, premature osteodegeneration, exacerbate progressive osteoporosis and increase the propensity towards vertebral degeneration, instability and deformation in humans.

**Methods:**

The influences of single-gene missense mutations, expressing either diabetes (*db/db*) or obese (*ob/ob*) metabolic syndromes on vertebral maturation and development in C57BL/KsJ mice were evaluated by radiological and macro-morphometric analysis of the resulting variances in osteodevelopment indices relative to control parameters between 8 and 16 weeks of age (syndrome onset @ 4 weeks), and the influences of low-dose 17-B-estradiol therapy on vertebral growth expression evaluated.

**Results:**

Associated with the indicative genotypic obesity and hyper-glycemic/-insulinemic states, both *db/db *and *ob/ob *mutants demonstrated a significant (P ≤ 0.05) elongation of total lumbar vertebrae column (VC) regional length, and individual lumbar vertebrae (LV1-5) lengths, relative to control VC and LV parameters. In contrast, LV1-5 width indices were suppressed in db/db and ob/ob mutants relative to control LV growth rates. Between 8 and 16 weeks of age, the suppressed LV1-5 width indices were sustained in both genotype mutant groups relative to control osteomaturation rates. The severity of LV1-5 width osteosuppression correlated with the severe systemic hyperglycemic and hypertriglyceridemic conditions sustained in *ob/ob *and *db/db *mutants. Low-dose 17-*B*-estradiol therapy (E2-HRx: 1.0 *ug*/ 0.1 ml oil s.c/3.5 days), initiated at 4 weeks of age (i.e., initial onset phase of *db/db *and *ob/ob *expressions) re-established control LV 1–5 width indices without influencing VC or LV lengths in db/db groups.

**Conclusion:**

These data demonstrate that the abnormal systemic endometabolic states associated with the expression of *db/db *and *ob/ob *genomutation syndromes suppress LV 1–5 width osteomaturation rates, but enhanced development related VC and LV length expression, relative to control indices in a progressive manner similar to recognized human metabolic syndrome conditions. Therapeutic E2 modulation of the hyperglycemic component of diabetes-obesity syndrome protected the regional LV from the mutation-induced osteopenic width-growth suppression. These data suggest that these genotype mutation models may prove valuable for the evaluation of therapeutic methodologies suitable for the treatment of human diabetes- or obesity-influenced, LV degeneration-linked human conditions, which demonstrate amelioration from conventional replacement therapies following diagnosis of systemic syndrome-induced LV osteomaturation-associated deformations.

## Background

Both Type 2 (NIDDM) diabetes and obesity represent dysregulated glucometabolic syndrome conditions in humans and experimental models [1-8]. The consequences of uncontrolled (non-corrected) hyperglycemic and hypertriglyceridemic systemic conditions have severe consequences on osteodevelopment and maturation indices, including suppressed skeletal development [1-3], increased incidence of osteopenia and osteoporosis [4-9], altered osteocytic proliferation rates [2,4,9,10], impaired bone healing potential [6], decreased osteoid tensile strength [1] and compromised osteomaturation indices [2,7,8,10-14]. Both diabetes (*db/*db) and obesity (*ob/*ob) syndromes in C57BL/KsJ mice are induced by inherited single-gene mutations, characterized by systemic and cellular hypercaloric Type II diabetes-obesity states [15-19]. These syndromes become expressed during the peripuberital period of life, at approximately 3–4 weeks of age, with subsequent progressive exacerbation of the syndrome conditions with age, similar to the progression from onset of youth [20] to adult maturity [21-23] human diabetes conditions. The resulting obese phenotypes [16-18] are related to a genetic mutation-induced leptin membrane receptor protein/ ligand misexpression (Table 1), generating hyperphagic experimental models which exhibit progressive body mass expansion, hyperglycemia, hyperinsulinemia, hypertriglyceridemia and premature cellular lipoinvolution, including skeletal osteopenia and chronic osteodegeneration [8,16-19,23-27]. The progressive, chronic metabolic compromise of cellular maturation and proliferation indices culminates in suppressed osteomaturation [28,29], cytostructural compromise[30], suppressed growth expression [1,29], premature osteoporosis [8,31-34], increased fracture susceptibility [34-38] and skeletal deformation [1-3,8].

Recent reports suggest that the severity of lumbar vertebral (LV) osteodegeneration and osteoporosis is enhanced in both diabetics and obese patients exhibiting blatant systemic syndrome aberrations accompanied by chronic lower back discomfort or deformation [3-5,8,11]. In general, conventional pharmacotherapeutic replacement regimes fail to completely ameliorate progressive LV deterioration in afflicted patients [8,10,31-38]. The lack of an identified experimental model that demonstrates progressive osteodegeneration following the expression of diabetes or obese states has impeded the evaluation of interventional therapeutic approaches focused on the alleviation of progressive LV osteodegeneration or destabilization [32-40]. The current studies were designed to evaluate the influences of diabetes (*db/*db) and obese (*ob/ob*) genotype-mutations towards the induction of VC and LV osteo-retardation and degeneration, recognized as chronic human syndrome complications [8,37], and the progressive influences of the syndrome conditions on LV morphometric and radiological indices associated with the duration of mutation expression in the C57BL/-KsJ murine model.

## Methods

### Animals

Adult, female C57BL/ KsJ mice (Jackson Laboratory, Bar Harbor, ME derived), between 4 and 16 weeks of age were used in these studies. Littermate controls (designated as mixed +/+ and +/? normal phenotypes/genotypes), as well as littermate diabetes (*db/db*) and obese (*ob/ob*)-mutant (homozygous recessive) genotypes (Table 1), were pair matched for phenotype, tissue sampling and systemic metabolic indices analyses (i.e., blood glucose, serum insulin and triglyceride concentrations) comparisons during the course of these studies (Table 2). All mice were housed five per cage, grouped according to genotype, under controlled environmental conditions (23 C), with an established photoperiod of 12 hr light/day (lights on: 0600 hr) [29]. Blood glucose levels (Ames Glucometer method), serum insulin and triglycerides levels [26,29] and body weights were monitored for each of the designated 8 and 16-week-old experimental age groups as previously described [26,29,41]. Animals exhibiting either lean (≤ 15 grams) or obese (≥ 25 grams) phenotypes (controls: ± 20 grams) and pronounced systemic hyperglycemia (≥ 200 mg/dl) relative to controls (≤150 mg/dl) by 8 weeks of age (Table 1) were considered as overt, Type 2 NIDDM obese (*ob/ob*) or diabetes (*db/db*) groups [16,36], with the continued expression of these indices denoted through the chronic 16-week old (Table 2) age group experimental periods.

### Radiographic and morphometric analysis of lumbar vertebral column (VC) and individual vertebrae (LV) maturation variances

At 8 and 16 weeks of age, each designated genotype group was subjected to light methoxyflurane (Parke-Davis, Detroit, MI) inhalation (45 sec exposure) anesthesia prior to radiographic imaging analysis of lumbar vertebral column (VC) regional length measurements, as well as individual lumbar vertebrae (LV) length and width measurements. A complete lumbar vertebral radiographic (1/30-1/24 sec exposure at a 40 KV/200 amp setting) image was captured on radiographic (Kodak, Rochester, NY) plate film, developed and the respective measurements of VC and individual LV 1–5 length and width indices determined using an Olympus flat-bed light-optics graphics recording unit connected to a data processing computer for statistical tabulation and analysis of morphometric measurement parameters [42]. All bone measurements were determined by enhanced digital image analysis of individual radiographs utilizing identified lumbar vertebral column location landmarks at both 8 and 16 weeks of age for all control and genotype-mutation designated groups. Morphometric data were collected, tabulated, analyzed and compared for intergroup differences relative to specified group mutation type (Table 1), age (duration) of mutation expression (Table 2) as well as LVC and LV variances.

### Estradiol treatments (E2-HRx)

17-*B*-estradiol (E2: 1 *ug*/ 3.5 days) was dissolved in sesame oil (0.1 ml) for subcutaneous injections (HRx: indicated as Day 0 of 3.5 day intervals) initiated at 3 weeks (21 days: weaning) of age. The oil (sesame: Sigma) vehicle (0.1 ml) served as the sham-control injection procedure as previously described [18,24]. These temporal dose regimes were selected based on previous studies indicating the restoration of diestral (i.e., control baseline) systemic ovarian steroid concentrations in hypogonadal *db/db *mutants following initiation of the E2-HRx therapies prior to the overt onset of the diabetes-associated syndrome [17,18].

### Statistical analysis

Values for body weights, bone morphometrics and systemic endometabolic indices were expressed as group means (± SEM) for the designated genotype groups. Inter-group and intra-group differences were determined using the Student's T-test exam, with a p ≤ 0.05 accepted as representing statistical differences for the specified parameter.

## Results

### Genotype (mutation)-related influences on body mass and systemic endometabolic indices

Between 8 and 16 weeks of age, control groups demonstrated stable body weights in association with systemic euglycemia, normoinsulinemia and basal circulating triglyceride (triacylglycerol) levels (Table 2). In contrast, both *db/db *and *ob/ob *mutation expressions induced significant increases in body masses and systemic endometabolic indices between 8 and 16 weeks of age relative to parameters (Table 2).

### Radiographic and morphometric analysis of LVC and LV 1–5 maturation variances associated with genotype mutation expression

**Figure 1:**
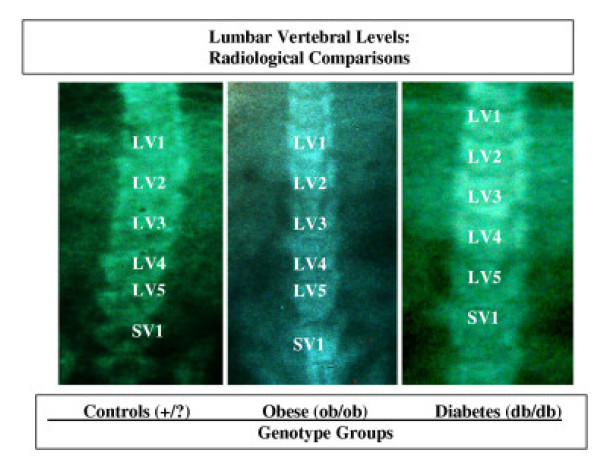
Radiological comparisons between the lumbar vertebral segments (LV 1–5) for control, obese (*ob/ob*) and diabetes (*db/db*) genotype-mutants groups are represented (x12.5) as indicators of length and width index parameters measured relative to the severity of diabetes-obesity syndrome aberrations for body weight and systemic endocrine/metabolic (Table 2) indices.

**Figure 2:**
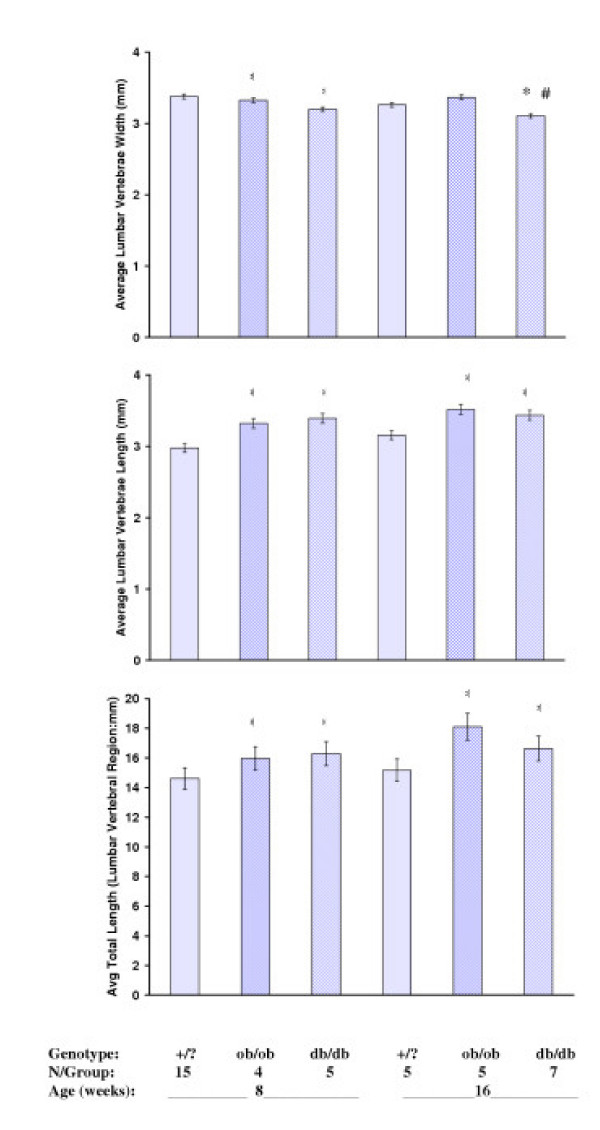
Representation of average lumber vertebral column (VC) lengths, as well as individual lumber vertebrae (LV) length and width indices, are denoted for control (+/?), obese (ob/ob) and diabetes (db/db) mutant genotypes as groups means (± SEM) at 8 and 16 weeks of age. Significant (P ≤ 0.05) intergroup differences (i.e., control v.s. genotype mutant type) at each designed age are denoted by asterisks (*). Age-dependent (i.e., 8 v.s. 16 week values for the same genotype) intragroup differences are denoted by #.

Radiographic (Figure 1) and morphometric (Figure 2) analysis of total LVC length and average LV 1–5 length and width indices demonstrated variances in VC maturation and growth indices associated with *db/db *and *ob/ob *mutation expressions in C57BL/KsJ groups. Compared to control growth indices (Figure 2), all genotype mutation groups demonstrated significant increases in average lumbar VC length measurements relative to control indices at 8 and 16 weeks of age (Figure 2) in association with hypercaloric endometabolic indices and expanded body masses (Table 2). By 16 weeks of age, the progressive and cumulative influences of the *db/db *and *ob/ob *mutation syndromes promoted exaggerated VC and LV lengths, but diminished individual LV 1–5 width expression, relative to control groups (Figure 2).

### Influences of 17-B-estradiol (E2) therapy (HRx) on VC and LV 1–5 growth indices in diabetes (*db/db*) genotype mutant groups

**Figure 3:**
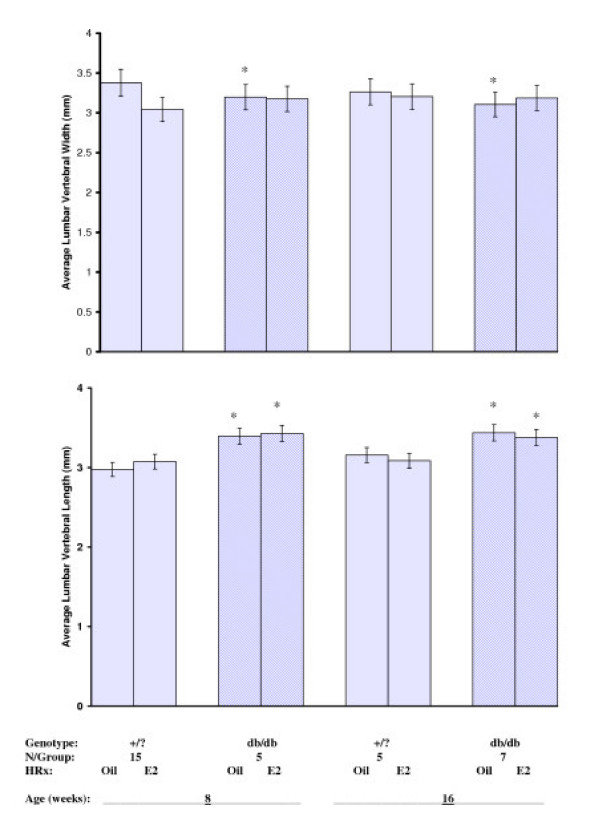
Influences on control (+/?) and diabetes (db/db)-mutant C57BL/KsJ group average lumbar vertebrae (LV) length and width indices following oil- or E2-HRx regimes at 8 and 16 weeks of age. All values are denoted as groups means (± SEM), with significant (P ≤ 0.05) intergroup (i.e. control v.s. diabetes genotypes for indicated HRx regimes) differences are denoted by asterisks (*)

The therapeutic (0.1 *ug*/0.1 ml oil vehicle: sc injection @ 3.5 day intervals) re-establishment of normoglycemia (Table 3) by the administered E2-HRx regime following expression of the *db/db *syndrome did not influence total VC length indices, or individual LV 1–5 length parameters, in the mutation group or in controls (Figure 3) at 8 weeks of age (i.e., 4 weeks post-onset of E2-HRx). In addition, at 16 weeks of age, the average LV 1–5 lengths of both oil- and E2-HRx *db/db *groups remained elongated relative to littermate control indices. In contrast, E2-HRx promoted a significant increase in average LV 1–5 width in the *db/db *mutant groups, relative to oil-HRx (sham vehicle injections) *db/db *mutants, and comparable to littermate controls (Figure 3) at both 8 and 16 weeks of age.

## Discussion

The results of the current studies demonstrate that the expression of diabetes and obese metabolic syndromes in C57BL/KsJ mice, attributable to the expression of single-gene mutations, compromises lumbar vertebral osteomaturation rates and individual LV 1–5 growth indices, culminating in variant VC development similar to chronic human syndrome complications [8,31,33,36,37,39]. The radiological and morphometric indications of pronounced VC and LV 1–5 length indices, contrasted with diminished LV 1–5 width parameters, in these experimental models are suggestive causes of the recognized increases of vertebral fracture, osteoporosis and torsion stress-induced dislocation susceptibilities in humans experiencing chronic diabetes or obesity-related systemic metabolic, endocrine or nutritional compromise [8,32-35,37,38]. The pronounced, progressive suppression of LV 1–5 width maturation, but pronounced individual LV and total VC regional elongation, indices occurred under the systemic influences of hyperglycemic, hyperinsulinemic and hyperlipidemic endocrine/metabolic stimulation. These data indicate that in these novel experimental models of dysregulated metabolic syndromes, variances in VC and LV growth expressions may be evaluated which are recognized to be shared with chronic human syndrome complications [8,37,39]. The restoration of normal LV 1–5 width indices in *db/db *mutants following E2-HRx indicates that correction of the glucometabolic disturbances with characterize the hypogonadal mutant model [28-30] re-establishes osteomaturation indices under persistent missense mutation (Table 1) expression influences [15,16]. These results indicate that the therapeutic re-establishment of a homeostatic systemic glucometabolic environment in *db/db *mutants supports the expression of normalized LV 1–5 width osteomaturation and VC stabilization, and counter-regulates the hyperglycemia-promoted susceptibilities to osteoporosis and VC destabilization resulting from chronic diabetes syndrome influences [1-5,8,18].

Multiple tissue types and cytometabolic expression events are recognized to be influenced by the systemic, interstitial and pericellular environmental aberrations which characterize glucometabolic, genotype mutation syndromes [4,8,16,18,23,33,37]. In addition to the intrinsic genome-directed influences on normal bone growth expression indices [2,3,5,6], the severity of expressed metabolic dysregulation is recognized to have a detrimental influence on cellular proliferation, metabolic homeostasis and osteostructural integrity, as well as the premature onset of cytoapoptosis, nuclear dissolution and subsequent organoinvolution [18,28-30]. As indicated for pancreatic [17], renal [43], hepatic [16,44], reproductive tract [28,29] and central nervous system [30,44-48] responses to dysregulated *db/db*-syndrome microenvironments, diabetes-affected cellular differentiation, proliferation and maturation indices are compromised, resulting in growth abnormalities and restricted lifespan expectancies [16,18,30,49]. In both the *db/db *and *ob/ob *groups, expression of the genotype mutations resulted in a severe hypercaloric, endocrine/metabolic disruption of normal VC and LV osteodevelopmental patterns [16,18]. These cytometabolic perturbations are recognized to be the result of the deleterious chronic, progressive influences on osteodevelopment [18,20,21], and have been associated with Type 2 (NIDDM) insulin resistance (insensitivity) in both animal models and humans exhibiting hyperglycemia and hyperinsulinemia [18,50-52]. Thus, in skeletal tissue affected by these cellular metabolic disturbances, altered growth expression rates would expectedly influence bone maturation (growth) indices as demonstrated by the *db/db *and *ob/ob *mutant groups. Associated with the continued duration of mutation expression (i.e. 8 to 16 weeks), the severity of skeletal compromise would be exacerbated [8,31,33] as cellular metabolic dysregulation [4,6,10,31,36,37] progressively expanded into uncompromised tissue areas. As in humans [8,11,32-34], the long-term exposure to metabolic compromise would gradually influence the integrity and structural stability of the osteoid matrix, decreasing tensile strength as a reflection of altered cytochemical composition [1,5,6,8,10,39,51,52] and viable cellular densities [2,4,6,10,13], increasing fracture susceptibility [35,38] and promoting chronic skeletal deformation [8,31,50]. The efficacy and mechanisms of action of E2 and related anti-osteoporosis therapeutic agents that exhibit systemic glucose normalization capabilities [10,24,25,39,40,52], under the continued deleterious influences of genotype mutation expressions, are currently being evaluated for osteo- maintenance/genesis properties.

## Conclusion

In summary, the current studies define the variable influences of *db/db *and *ob/ob *genotype mutations on VC and LV 1–5 osteomaturation indices, and the structural compromise in LV width indices promoted by these dysregulated expression syndromes. Of particular interest were the structural elongation of VC and LV 1–5 osteomaturation parameters, and LV 1–5 width restrictions, in *db/db *and *ob/ob *mutants associated with the hypercaloric metabolic state that characterized both mutation syndromes. Although altered vertebral width expression occurred under such aberrant metabolic syndrome conditions, the therapeutic normalization of systemic glucose concentrations by E2-HRx stimulated LV osteodevelopment (width indices) comparable to control parameters. The chronic influences of these metabolic syndromes on progressive VC and LV osteodevelopment are regarded as intrinsic components of expressed skeletal compromise associated with altered bone cellular proliferation rates, density, cytochemical composition and diminished skeletal strength exhibited by both humans and experimental models exhibiting similar dysregulated metabolic syndromes [16-18,25,27]. The results of these studies suggest that these genetic models may be suitable for the further evaluation of novel manipulative or therapeutic treatments for osteomoderating events which are expressed in similar dysregulated metabolic-based human syndromes.

## Competing interests

The author(s) declare that they have no competing interests.

## Authors' contributions

The authors shared all responsibilities related to the collection and analysis of data associated with these studies.

## Pre-publication history

The pre-publication history for this paper can be accessed here:



## Figures and Tables

**Table 1:** Comparison of genotype mutation-related deficiencies in C57BL/KsJ Mice

**Genotype**	**Mutation**	**Characteristic**	**Syndrome Indices**
(*+/?*)	Controls (Heterozygous Littermates)	None	Normal
(*db/db*)	Diabetes (Lepr^*db*^)	Leptin Membrane Receptor (lf) Missense Mutation Defect (leptin insensitive)	Obese, Type 2 NIDDM (severe hyperglycemia & hyperinsulinemia)
(*ob/ob*)	Obese (Lep^ob^)	Leptin Protein Deficient (leptin gene mutation; leptin sensitive)	Obese, Type 2NIDDM (Moderate glycemia & insulin insensitive)

Comparison between genotypic indices induced by the indicate mutations, relative to normal littermate controls, and the attributed protein expression deficits associated with metabolic syndrome expression complications when expressed on the C57BL/KsJ murine background strain. An age-related exacerbation of the severity of mutation-induced metabolic/structural complications occurs for each genotype mutation.

**Table 2:** Comparisons between phenotypic and systemic endocrine/metabolic indices Induced by expression of diabetes (*db/db*) and obese (*ob/ob*) mutations in C57BL/KsJ mice

Parameter	Age(weeks)	Controls	Diabetes	Obese
Body Weight (g)	8	21.1 ± 0.3 (16)	*33.7 ± 0.8 (16)	*37.0 ± 1.3 (16)
	16	25.2 ± 0.5 (12)	*41.0 ± 2.4 (8)	*59.3 ± 1.2 (10)
Blood Glucose (mg/dl)	8	108.6 ± 7.7 (12)	*278.5 ± 22.6 (12)	*178.9 ± 15.4 (12)
	16	128.3 ± 7.9 (8)	*356.6 ± 37.6 (8)	*150.9 ± 5.8 (8)
Serum Insulin (pg/ml)	8	1184.3 ± 121.4 (12)	*3862.8 ± 231.7 (12)	*2342.9 ± 186.4 (12)
	16	1386.0 ± 132.0 (8)	*3300.0 ± 181.0 (8)	*2000.0 ± 150.0 (8)
Serum Triglycerides (mg/ml)	8	162.0 ± 18.0 (12)	*300.0 ± 50.0 (12)	*300.0 ± 53.0 (12)
	16	162.0 ± 24.0 (8)	*300.0 ± 83.0 (8)	*300.0 ± 43.0 (8)

Comparisons between indicated phenotype and systemic endocrine/metabolic parameters in control, diabetes- or obese-mutant C57BK/KsJ mice at 8 and 16 weeks of age are demoted as group means (± SEM) for the designated number (N) of animals per group. Significant (P ≤ 0.05) intergroup differences (i.e. control v.s. genotype group values) at the designated age are indicated by asterisks (*).

**Table 3:** Influences of E2-HRx on body weights and blood glucose levels in control and diabetes-mutant C57BL/KsJ mice

Group	Age (weeks)	HRx	Parameters	
			Body Weight(g)	Blood Glucose(mg/dl)
Control	8	Oil	21.1 ± 0.3	109 ± 8
		E2	21.8 ± 2	118 ± 8
Diabetes	8	Oil	33.7 ± 0.8*	278.5 ± 23*
		E2	34.2 ± 0.7*	261.1 ± 16*
Control	16	Oil	25.2 ± 0.5	128.3 ± 8
		E2	24.2 ± 0.5	113.3 ± 9
Diabetes	16	Oil	41.0 ± 3.4*	356.6 ± 38*
		E2	44.1 ± 1.8*	138.3 ± 20^#^

Comparisons between indicated phenotype and systemic endocrine/metabolic parameters in control, diabetes- or obese-mutant C57BK/KsJ mice at 8 and 16 weeks of age are demoted as group means (± SEM) for the designated number (N) of animals per group. Significant (P ≤ 0.05) intergroup differences (i.e. control v.s. genotype group values) at the designated age are indicated by asterisks (*).
